# CD22 blockade aggravates EAE and its role in microglia polarization

**DOI:** 10.1111/cns.14736

**Published:** 2024-05-13

**Authors:** Weiwei Xiang, Kan Wang, Lu Han, Ze Wang, Zhiyang Zhou, Shuwei Bai, Jing Peng, Chong Xie, Yangtai Guan

**Affiliations:** ^1^ Department of Neurology Renji Hospital, Shanghai Jiao Tong University School of Medicine Shanghai China; ^2^ Institute of Reproduction and Development, Obstetrics & Gynecology Hospital Fudan University Shanghai China; ^3^ Shanghai Key Laboratory of Reproduction and Development Shanghai China; ^4^ Department of Neurology Shanghai Punan Hospital Shanghai China

**Keywords:** CD22, EAE, microglia, multiple sclerosis, Siglec

## Abstract

**Aims:**

Multiple sclerosis (MS) is a neuroinflammatory demyelinating disease. Microglia are reportedly involved in the pathogenesis of MS. However, the key molecules that control the inflammatory activity of microglia in MS have not been identified.

**Methods:**

Experimental autoimmune encephalomyelitis (EAE) mice were randomized into CD22 blockade and control groups. The expression levels of microglial CD22 were measured by flow cytometry, qRT–PCR, and immunofluorescence. The effects of CD22 blockade were examined via in vitro and in vivo studies.

**Results:**

We detected increased expression of microglial CD22 in EAE mice. In addition, an in vitro study revealed that lipopolysaccharide upregulated the expression of CD22 in microglia and that CD22 blockade modulated microglial polarization. Moreover, an in vivo study demonstrated that CD22 blockade aggravated EAE in mice and promoted microglial M1 polarization.

**Conclusion:**

Collectively, our study indicates that CD22 may be protective against EAE and may play a critical role in the maintenance of immune homeostasis in EAE mice.

## Graphical abstract

Microglial CD22 was upregulated following inflammation. CD22 blockade exacerbated EAE in mice and induced the polarization of microglia toward the M1 phenotype.

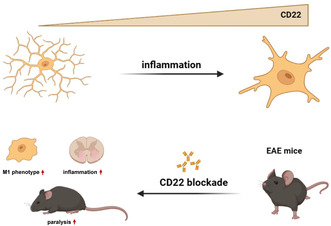

## 1 INTRODUCTION

Multiple sclerosis (MS) is an autoimmune disease of the central nervous system (CNS),^1^, ^2^ and experimental autoimmune encephalomyelitis (EAE) mimics the typical pathologies of MS including inflammation and demyelinating and glial hyperplasia, and induces typical behavioral manifestations.^3^, ^4^

Microglia are a group of glial cells with multiple functions that are widely distributed in the CNS.^5^ The pathogenesis of EAE and MS involves microglia.^6^, ^7^, ^8^, ^9^, ^10^, ^11^ The effector functions of microglia in EAE mice can be divided into four categories: phagocytosis, synaptic pruning, antigen presentation, and secretion of pro‐ and anti‐inflammatory mediators, and these functions can be both neuroprotective and harmful.^12^ Recently, there has been renewed interest in identifying the key molecules that control the inflammatory activity of microglia.

Sialic acid‐binding immunoglobulin‐like lectin‐2 (Siglec‐2), also known as CD22, is predominantly expressed in B cells.^13^, ^14^, ^15^ However, recent studies have shown elevated expression of CD22 in the CNS of Alzheimer's disease (AD) and Niemann–Pick disease patients.^16^, ^17^ Moreover, researchers found that CD22 was upregulated on aged microglia, and blocking CD22 improved the cognitive function of aged animals.^18^

To date, little is known about whether CD22 may affect neuroinflammation following MS. In the present study, we aimed to determine the expression level of microglial CD22 following inflammation and EAE. In addition, we studied the role of CD22 in microglial activity and neurological deficits after EAE and the underlying mechanisms involved.

## 2 METHODS

### 2.1 Animals

The animals used in this experiment were 6–8‐week‐old C57BL/6 mice weighing 18–20 g. The mice were reared at room temperature (20–25°C) with a 12‐h cycle of light and darkness. All animal experiments strictly followed the guidelines of the Shanghai Model Organisms Center's Ethical Committee in Animal Research.

### 2.2 Induction of EAE and treatment

The EAE model was constructed as previously reported^19^: MOG_35‐55_ (GL Biochem Ltd., Shanghai, China) was dissolved in PBS and then mixed with complete Freund's adjuvant (8 mg/mL) (strain H37RA; Difco, USA) at a 1:1 volume ratio. Female mice were injected with the emulsified solution at four sites. Two intraperitoneal injections of pertussis toxin (200 ng/mouse) (Millipore, Billerica, MA, USA) were given at 0 and 2 days after immunization. Mice were clinically scored by two researchers following the double‐blind principle and recorded as follows: tail weakness, 0.5; tail paralysis, 1; faltering gait with hind limb weakness, 2; bilateral hind limb paralysis, 3; forelimb weakness, 4; and near death, 5. Mice were administered an anti‐CD22 monoclonal antibody (mAb) (CY34.1, BioXcell) or an IgG control via the tail vein at a dose of 400 μg/20 g from Day 4 every 2 days.

### 2.3 Cultures of BV2 cells and treatment

BV2 cells were seeded in six‐well plates at a density of 200,000/well, and lipopolysaccharide (LPS) was added after the cells were adherent. After 24 h, BV2 cells were collected for qPCR and flow cytometry. To detect the effect of CD22 blockade on the polarization of BV2 cells, BV2 cells were pretreated with 10 μg/mL CD22 mAb (CY34.1, BioXcell) and control IgG for 2 h, followed by treatment with 100 ng/mL LPS. The cells were harvested for subsequent experiments after 24 h.

### 2.4 Histological analysis

Lumbar enlargements were harvested for histological analysis. After fixation with 4% paraformaldehyde, the tissue was cut into 5‐μm‐thick sections. As reported previously,^19^ hematoxylin and eosin (H&E) staining was carried out to assess the infiltration of inflammatory cells, while Luxol fast blue staining (LFB) staining was carried out to assess the severity of demyelination.

### 2.5 Immunofluorescence

The tissue was cut into 10‐μm‐thick sections. After washing with PBS, the slices were incubated with Quick Block (Beyotime Shanghai, China) for 10 min. Next, the slices were incubated with multiple primary antibodies for 12 h at 4°C, and then incubated with the appropriate secondary antibodies. The slices were also incubated with DAPI for 5 min at room temperature to show the nucleus. The primary antibodies used included rabbit anti‐Iba1 (1:1000, Wako), rat anti‐Iba1 (1:100, Abcam), rat anti‐CD22 (1:1000, Abcam), rabbit anti‐CD206 (1:1000, Abcam), and rabbit anti‐iNOS (1:400, Abcam). Finally, images were captured using a Leica fluorescence microscope and analyzed by ImageJ.

### 2.6 qRT–PCR

Total RNA was extracted from BV2 cells with TRIzol reagent. RNA was reversely transcribed into cDNA following the manufacturer's protocols (Vazyme, Shanghai, China). The expression level of mRNA was quantified using a real‐time PCR detection system with SYBR qPCR Master Mix (Vazyme, Shanghai, China). The sequences of primers used were as follows: *Gapdh* forward, 5′‐GGTTGTCTCCTGCGACTTCA‐3′ and reverse, 5′‐TGGTCCAGGGTTTCTTACTCC‐3′; *Cd206* forward, 5′‐GTCTGAGTGTACGCAGTGGTTGG‐3′ and reverse, 5′‐ TCTGATGGACTTCCTGGTAGCC‐3′; *Inos* forward, 5′‐ATCTTGGAGCGAGTTGTGGATTGTC‐3′ and reverse, 5′‐TAGGTGAGGGCTTGGCTGAGTG‐3′; and Cd22 forward, 5′‐CCACTCCTCAGGCCAGAAACT3′ and reverse, 5′‐TGCCGATGGTCTCTGGACTG‐3′.

### 2.7 Western blots

EAE mice were sacrificed on Day 17 postimmunization. The spinal cords were harvested and homogenized. After centrifugation at 12,000×*g* for 15 min, the supernatant was aspirated. Then, 5X loading buffer was added, and the protein was boiled in a 100°C metal bath for 10 min to denature. The protein was separated by SDS–PAGE and transferred onto a PVDF membrane (Merck KGaA, Darmstadt, Germany). After blocking, the membranes were incubated with primary antibodies against fibrinogen (1:1000, Abcam), iNOS (1:1000, Abcam), and GAPDH (1:10000, Abcam). Subsequently, the membranes were washed with TBST and incubated with an HRP‐labeled secondary antibody (1:10000, Jackson Immuno). The membranes were imaged on a Bio‐Rad (Bio‐Rad, Hercules, CA), and the image information was analyzed with ImageJ.

### 2.8 Flow cytometry

The brains and spinal cords were collected and placed in cold HBSS. The tissue was cut with sharp scissors and spun at 500 × g for 5 min. The tissue pellets were resuspended in digestion buffer and incubated in a 37°C water bath for 30 min. Then, the tissue was homogenized with 70 μm nylon cell strainers. The tissue pellets were collected, resuspended in 40% Percoll, and centrifuged at 650×*g* for 25 min at room temperature. The cell pellets were resuspended in PBS and stained following the manufacturer's instructions. All antibodies used in this study were purchased from Biolegend. Flow cytometry was performed on FACS celesta (BD Bioscience), and the data were analyzed with FlowJo software.

### 2.9 Assessment of vascular integrity by analysis of EB extravasation

Animals were injected with 2% Evans blue (EB) (5 mL/kg). Four hours later, the mice were sacrificed, and the spinal cords were homogenized in 0.5 mL of 50% trichloroacetic acid solution. The samples were centrifuged at 12,000×*g* for 20 min to extract the EB. The supernatant was diluted 1:3 with 100% ethanol. The amount of EB was quantified by a spectrophotometer at 610 nm.

### 2.10 Cytometric bead array

The spinal cords were homogenized and centrifuged at 5000×*g* for 5 min. The supernatant was aspirated, and the concentration of inflammatory cytokines was measured using a cytometric bead array (CBA) (Biolegend) following the manufacturer's instructions. CBA samples were run on a FACS celesta (BD Bioscience) and analyzed following the manufacturer's instructions.

### 2.11 Data analysis and statistics

All the data were analyzed for normality by the Shapiro–Wilk normality test. Student's *t*‐test or the Mann–Whitney *U*‐test was used for the analysis of parametric data and nonparametric data, respectively. For multigroup comparisons, one‐way ANOVA or the Kruskal–Wallis test was used to compare the differences between groups. Spearman correlation analysis was used to measure the monotonic relationship between the two variables X and Y. All the data are presented as the means ± SEMs, and the statistical analysis was performed using GraphPad Prism (v.8.0) software. A *p*‐value < 0.05 was considered to indicate a significant difference.

## 3 RESULTS

### 3.1 Upregulation of CD22 in microglia following inflammation and EAE

**FIGURE 1:**
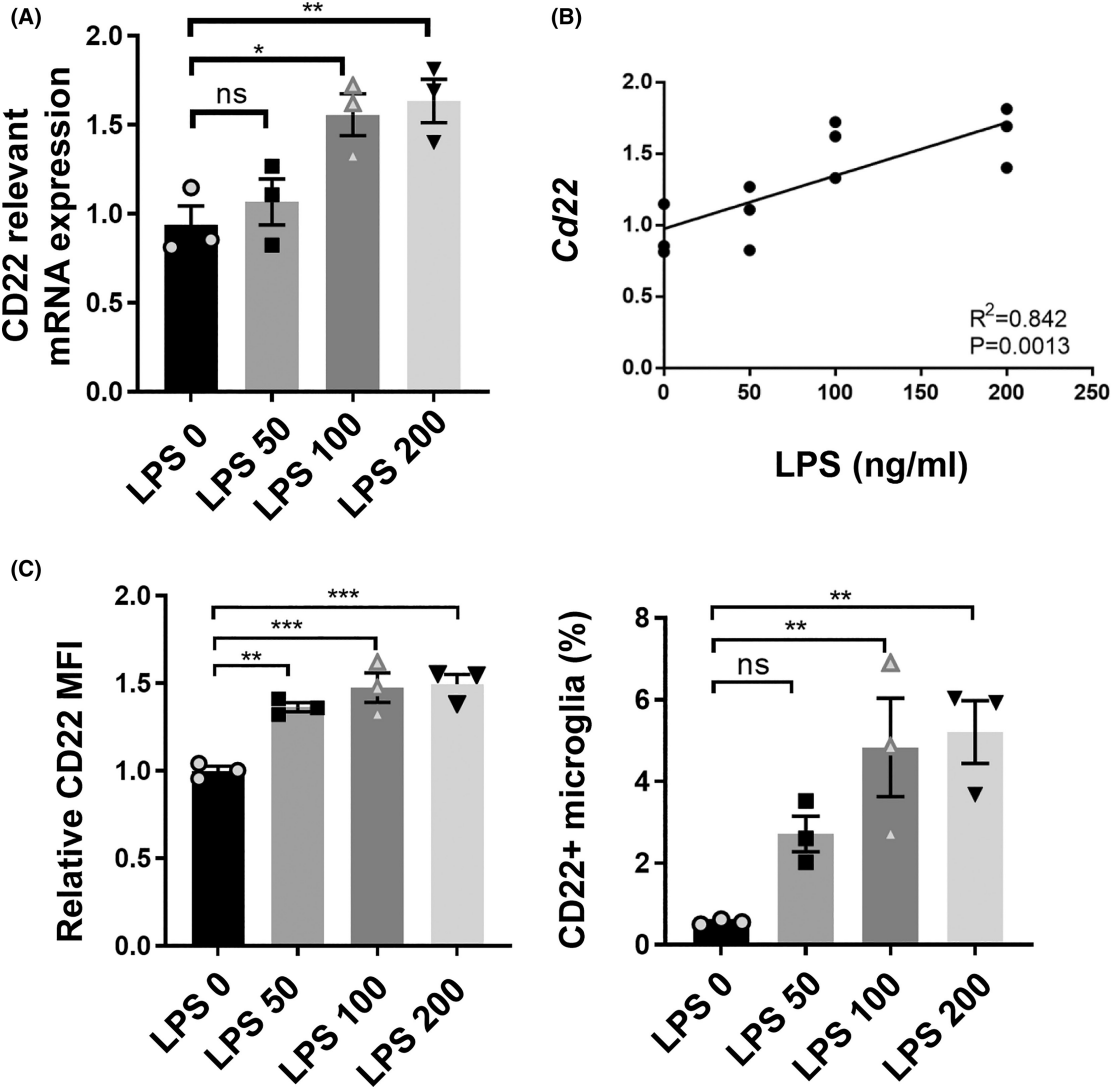
LPS induced an increase in CD22 expression in BV2 cells. (A) The relative mRNA expression level of CD22 in BV2 cells treated with LPS; (B) correlation analysis of the LPS concentration and relative mRNA expression level of CD22 (*R* ^2^ = 0.842, *p* = 0.0013); (C) statistical analysis of the relative MFI of CD22 and the percentage of CD22^+^ microglia; the data are presented as the means ± SEMs; *n* = 3 in each group; all the data were analyzed for normality by the Shapiro–Wilk normality test; differences between four groups were compared with one‐way ANOVA, and Dunnett's test was used for multiple comparisons; ns indicates no significant difference; *p < 0.05; **p < 0.01; ***p < 0.001.

We treated BV2 cells with 0 ng/mL, 50 ng/mL, 100 ng/mL, and or 200 ng/mL LPS for 24 h; then, we harvested the cells and detected the expression level of CD22. As shown in Figure 1A–C, we found markedly increased expression of CD22 in BV2 cells. In addition, LPS dose‐dependently induced the expression of CD22.

**FIGURE 2:**
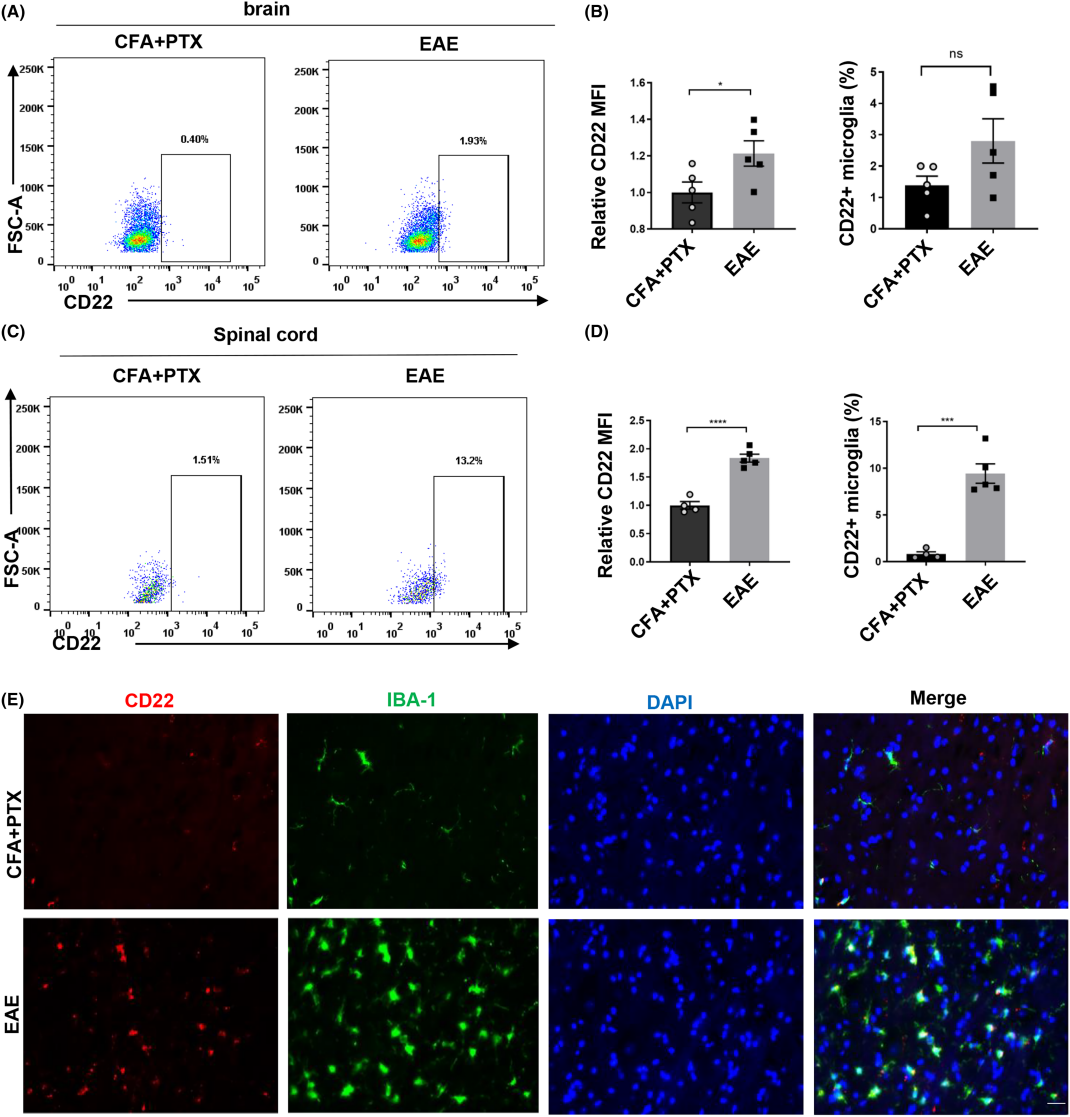
Microglial CD22 was upregulated in EAE mice. (A, C) Flow cytometry analysis of the percentage of CD22^+^ microglia in EAE and CFA + PTX mice; (B, D) statistical analysis of the relative MFI of CD22 and the percentage of CD22^+^ microglia; (E) immunofluorescence staining of DAPI (blue), CD22 (red), and Iba‐1 (green), scale bar = 50 μm; the data are presented as the means ± SEMs; *n* = 4–5 in each group; all the data were analyzed for normality by the Shapiro–Wilk normality test; Student's *t*‐test was used for the analysis of data in A–D; ns indicates no significant difference; *p < 0.05; ***p < 0.001; ****p < 0.0001.

To determine the expression of microglial CD22 following EAE, we sacrificed the mice at the peak of onset and performed flow cytometry. We found that the level of microglial CD22 was slightly increased in the brain tissue of EAE mice but was markedly increased in the spinal cords of EAE mice (Figure 2A–D). In addition, we used immunofluorescence to confirm the expression of CD22 in the spinal cords of EAE mice. Consistent with the flow cytometry results, we found that the expression of microglial CD22 was upregulated in the EAE mice compared to that in the control mice (Figure 2E). Collectively, these data indicated that the expression of microglial CD22 was upregulated following inflammation and EAE.

### 3.2 CD22 blockade induced the polarization of BV2 cells toward the M1 phenotype

**FIGURE 3:**
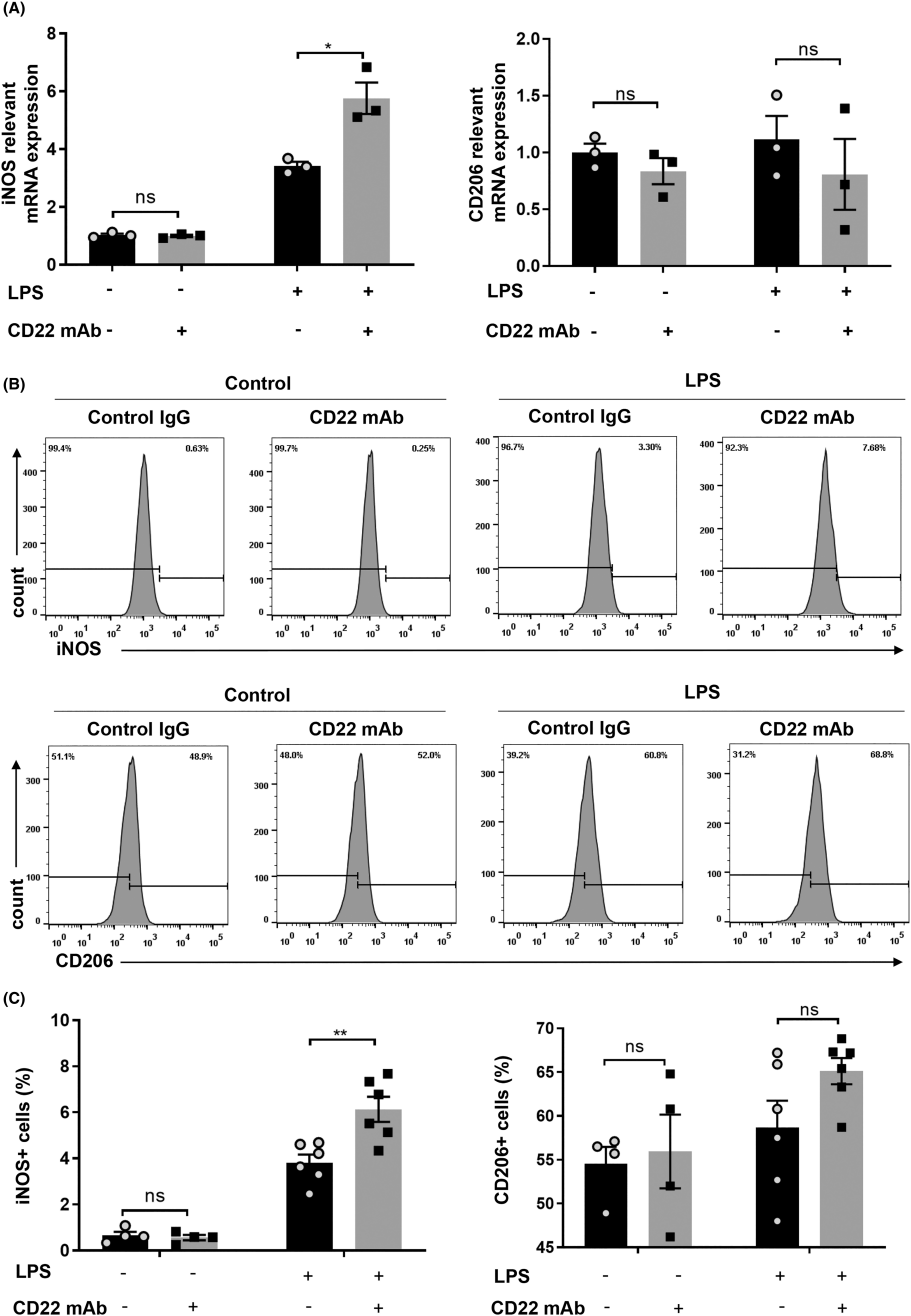
CD22 blockade induced polarization of BV2 cells toward the M1 phenotype. (A) The mRNA expression levels of iNOS and CD206, *n* = 3 in each group; (B) the histogram curve of iNOS and CD206; (C) statistical analysis of the percentage of iNOS^+^ or CD206^+^ cells, *n* = 4–6 in each group; the data are presented as the means ± SEMs; all the data were analyzed for normality by the Shapiro–Wilk normality test; Student's *t*‐test was used for the analysis of data in A–C; ns indicates no significant difference; *p < 0.05; **p < 0.01.

To detect the effect of CD22 on microglial polarization following inflammation, LPS‐treated BV2 cells were treated with 10 μg/mL anti‐CD22 mAb, and the expression of iNOS and CD206 was detected via qRT–PCR and flow cytometry. As shown in Figure 3A–C, the anti‐CD22 mAb significantly promoted the expression of M1‐related markers in BV2 cells but had no significant effect on the expression of M2‐related markers.

### 3.3 CD22 blockade exacerbated the clinical course and pathological changes in EAE mice

**FIGURE 4:**
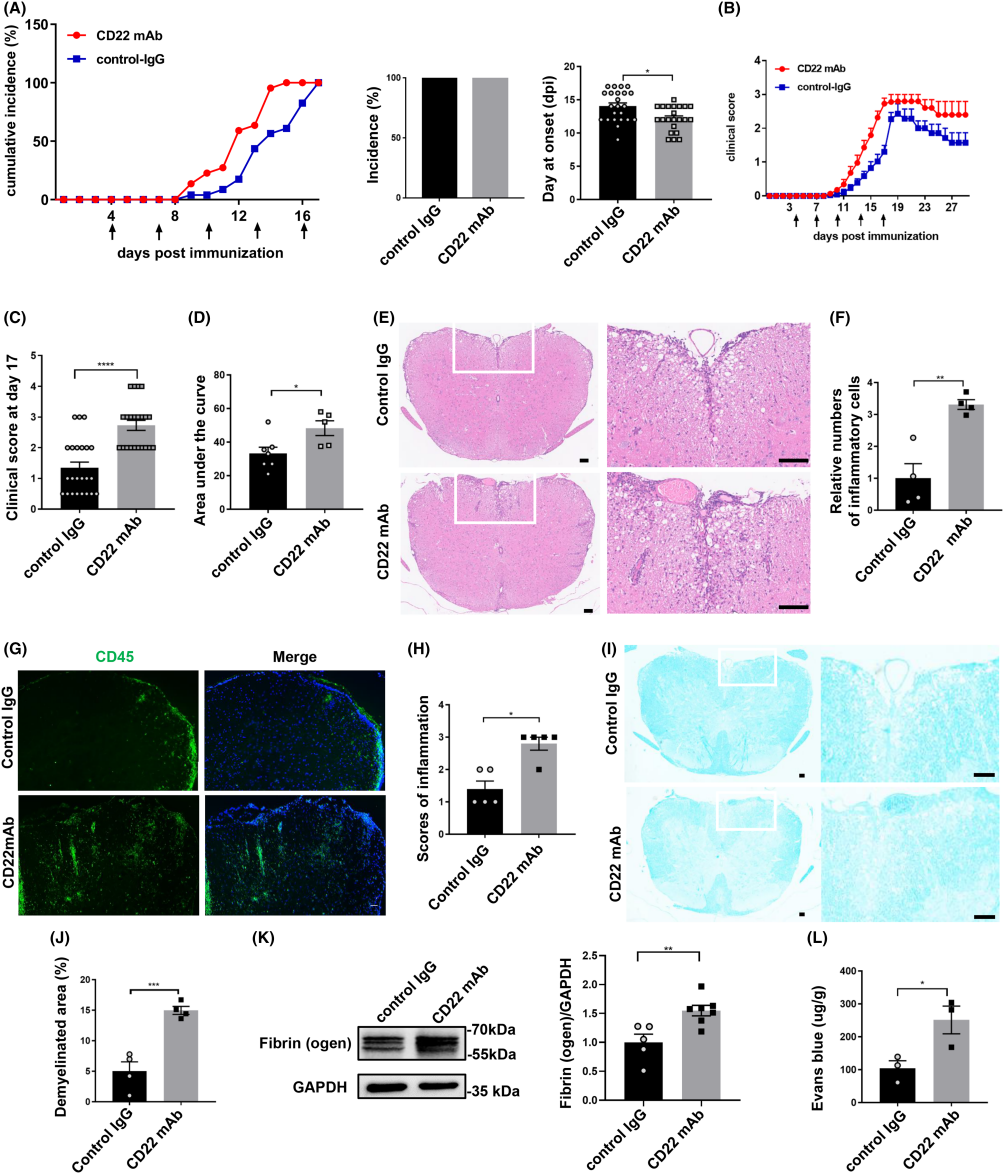
CD22 blockade exacerbated EAE. (A) Cumulative incidence over time in groups of CD22 mAb‐injected EAE mice and control IgG‐injected EAE mice and statistical analysis of disease onset time between the two groups, *n* = 22–23; (B) curve of clinical scores over time in CD22 mAb‐injected EAE mice and control IgG‐injected EAE mice; (C) statistical analysis of clinical scores on Day 17 in EAE mice receiving CD22 mAb or control IgG, *n* = 22–23; (D) statistical analysis of the area under the curve between the two groups of mice, *n* = 5–7; (E, F) H&E staining of intact spinal cord sections and statistical analysis of inflammatory cell infiltration, scale bar = 100 μm, *n* = 4 in each group; (G) immunostaining of DAPI (blue) and CD45 (green), scale bar = 50 μm; (H) statistical analysis of the score of inflammation, *n* = 5 in each group; (I, J) LFB staining of intact spinal cord sections and statistical analysis of demyelinating area, scale bar = 100 μm, *n* = 4 in each group; (K) the expression level of fibrinogen in spinal cord sections as measured by Western blot detection in EAE mice receiving CD22 mAb or control IgG, and the statistical analysis of Western blot detection, *n* = 5–7; (L) quantitative analysis of EB in EAE mice receiving CD22 mAb or control IgG, *n* = 3 in each group; all the data were analyzed for normality by the Shapiro–Wilk normality test; Student's *t*‐test was used for the analysis of data in D–F, I–L. Mann–Whitney *U*‐test was used for the analysis of data in A–C, G–H; the data are presented as the means ± SEMs; *p < 0.05; **p < 0.01; ***p < 0.001; ****p < 0.0001.

To study the functional significance of CD22 in vivo, we injected a CD22‐blocking antibody or control IgG into EAE mice via the tail vein. We first assessed the behavioral changes of these mice in both groups by clinical scores. Compared with that of the control IgG group, the average onset time of EAE mice injected with the CD22 mAb was significantly earlier (Figure 4A). In addition, CD22 blockade significantly exacerbated the clinical symptoms of EAE mice (Figure 4B–D).

As inflammatory cell infiltration and demyelination are the most typical pathological features in EAE mice, we performed H&E staining and LFB staining to study inflammatory infiltration and demyelination, respectively, in the spinal cords of EAE mice. We detected inflammatory cell infiltration in both groups of mice, and compared with that in the control group, the number of inflammatory cells was significantly greater in the EAE group receiving the anti‐CD22 mAb (Figure 4E,F). Immunostaining of CD45 also revealed similar results (Figure 4G,H). In addition, anti‐CD22 mAb treatment increased demyelination in EAE mice (Figure 4I,J).

Given that destruction of the blood–brain barrier (BBB) is another typical pathological feature in EAE mice, we performed a BBB permeability assay to evaluate BBB integrity. Fibrinogen is a glycoprotein synthesized and secreted by liver cells.^20^, ^21^ Fibrinogen cannot be detected in the CNS due to the presence of the BBB in the physiological state.^20^, ^21^ However, fibrinogen leaks into the CNS after destruction of the BBB. Western blot analysis revealed that treatment with the CD22 mAb significantly increased the level of fibrinogen in the spinal cords of EAE mice (Figure 4K). In addition, EB extravasation was also measured to evaluate BBB permeability after anti‐CD22 mAb treatment. EB quantification revealed that the amount of EB leakage was greater in EAE mice treated with the anti‐CD22 mAb than in control mice (Figure 4L). Taken together, these results indicated that the anti‐CD22 mAb increased inflammatory cell infiltration, demyelination, and destruction of the BBB in EAE mice.

### 3.4 CD22 blockade activated microglia and promoted microglial M1 polarization in EAE mice

**FIGURE 5:**
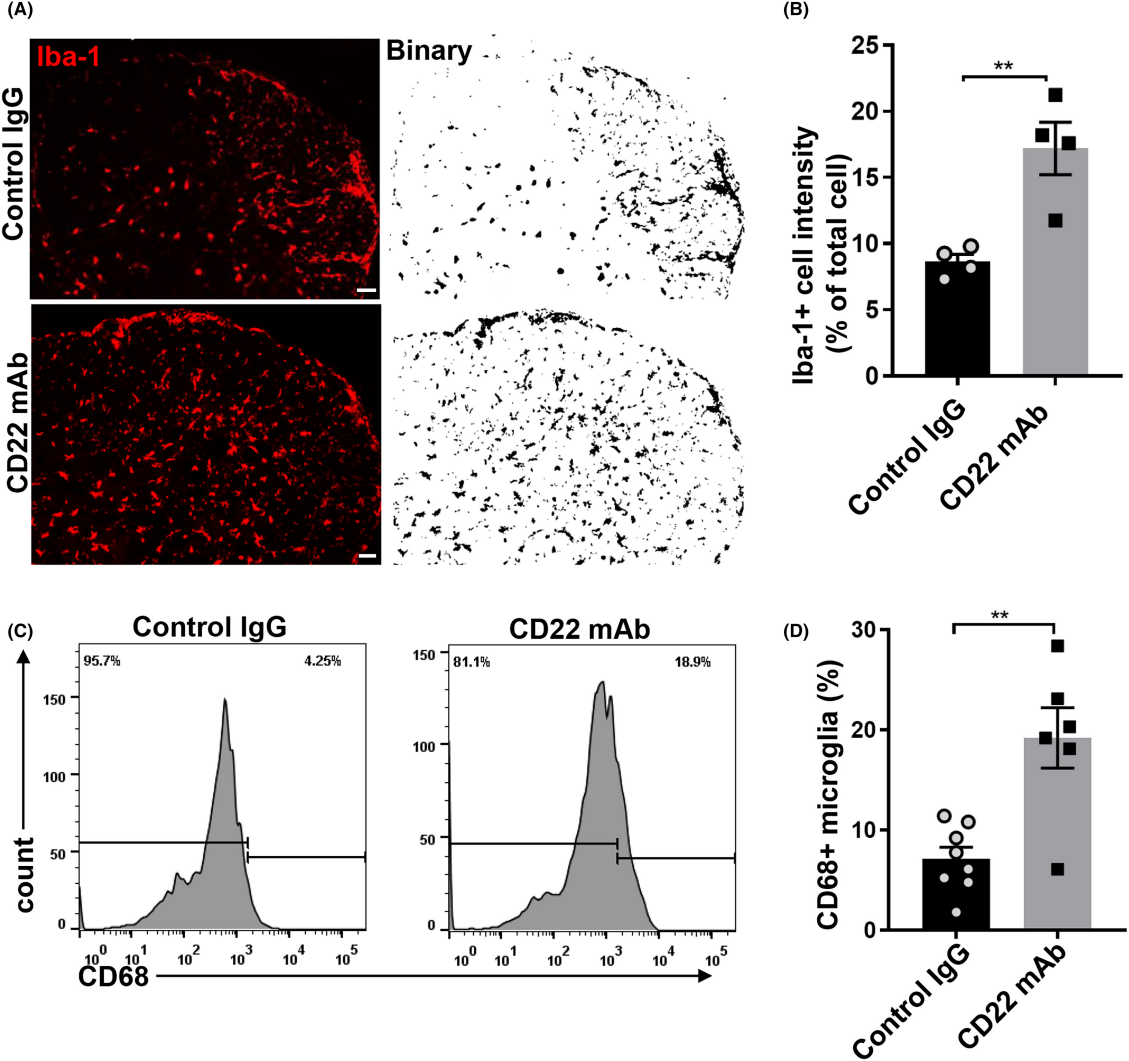
Microglia were activated in EAE mice receiving the CD22 mAb. (A) Immunostaining of Iba‐1 (red) and representative binary image of EAE mice receiving CD22 mAb or control IgG, scale bar = 50 μm; (B) statistical analysis of the percentage of Iba‐1^+^ cell relative to total cells, *n* = 4 in each group; (C) histogram curve of CD68 expression in microglia using flow cytometry; (D) statistical analysis of the difference in the percentage of CD68^+^ microglia relative to the total microglia, *n* = 6–8 in each group; all the data were analyzed for normality by the Shapiro–Wilk normality test; Student's *t*‐test was used for the analysis of data in A–D; the data are presented as the means ± SEMs; **p < 0.01.

To test the effect of the anti‐CD22 mAb on microglia in vivo, we first examined the activation of microglia by immunostaining and flow cytometry. As shown in Figure 5A–D, the number of Iba1^+^ cells and the percentage of CD68^+^ microglia in EAE mice treated with the anti‐CD22 mAb were significantly greater than those in the control group, which indicated the activation of microglia.

**FIGURE 6:**
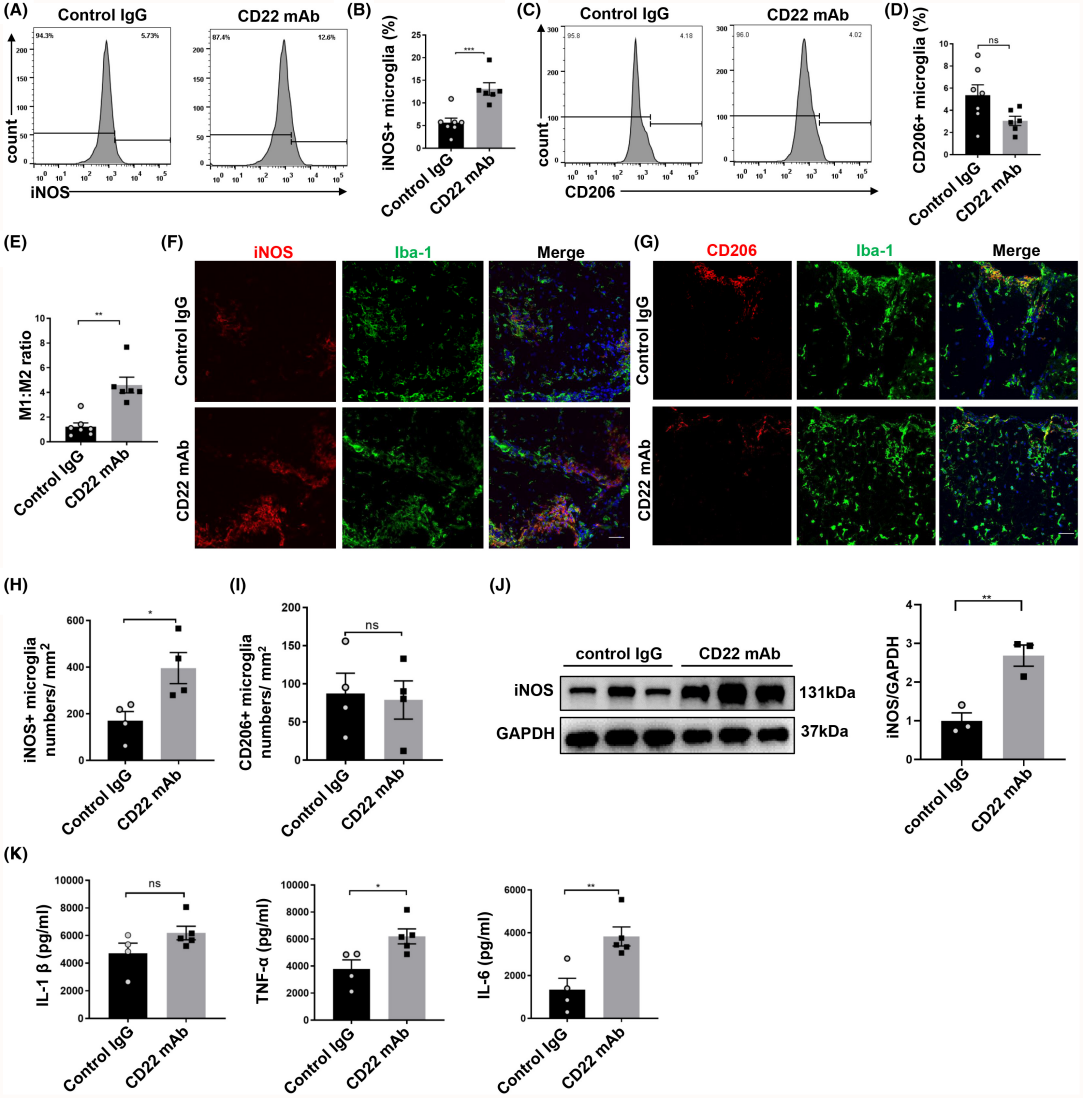
CD22 blockade increased iNOS^+^ microglia and the expression levels of M1 phenotype‐related cytokines in EAE mice. (A, C) The histogram curve of iNOS and CD206 expression in microglia using flow cytometry; (B, D) statistical analysis of the difference in the percentage of iNOS^+^ and CD206^+^ microglia relative to the total microglia, *n* = 6–8 in each group; (E) statistical analysis of the ratio of M1/M2 in EAE mice receiving control IgG or CD22 mAb, *n* = 6–8 in each group; (F, G) immunofluorescence staining of DAPI (blue), Iba‐1 (green), iNOS and CD206 (red) in EAE mice receiving CD22 mAb or control IgG, scale bar = 50 μm; (H, I) statistical analysis of the number of iNOS^+^ and CD206^+^ microglia in spinal cord sections of EAE mice receiving CD22 mAb or control IgG, *n* = 4 in each group; (J) the expression level of iNOS in spinal cord sections as measured by Western blot detection in EAE mice receiving CD22 mAb or control IgG, and the statistical analysis of Western blot detection, *n* = 3; (K) levels of the proinflammatory cytokines in EAE mice receiving CD22 mAb or control IgG, *n* = 4–5 in each group; all the data were analyzed for normality by the Shapiro–Wilk normality test; Student's *t*‐test was used for the analysis of data in A–D, F–K. Mann–Whitney *U*‐test was used for the analysis of data in E; the data are presented as the means ± SEMs; ns indicates no significant difference; *p < 0.05; **p < 0.01; ***p < 0.001.

As mentioned above, we found that CD22 blockade could promote microglial M1 polarization in vitro, but whether CD22 blockade could affect the polarization of microglia in EAE mice warrants further tests. As shown in Figure 6A,B, the percentage of iNOS^+^ microglia was significantly greater in EAE mice treated with the anti‐CD22 mAb than in control mice. However, there was no significant difference in the percentage of CD206^+^ microglia between the two groups (Figure 6C,D). Moreover, the M1/M2 ratio was also significantly greater in EAE mice receiving the anti‐CD22 mAb than in those receiving control IgG (Figure 6E). Consistently, immunostaining also revealed an increased number of iNOS^+^ microglia in EAE mice treated with the anti‐CD22 mAb, while there was no significant difference in the number of CD206^+^ microglia between the two groups (Figure 6F–I). Western blot analysis also revealed that treatment with the CD22 mAb significantly increased the level of iNOS in the spinal cords of EAE mice **(**Figure 6J). In addition, as shown in Figure 6K, CD22 blockade increased the levels of the proinflammatory cytokines interleukin (IL)‐1β, IL‐6, and tumor necrosis factor (TNF)‐α in EAE mice compared to those in control mice, but the differences in the levels of IL‐1β were not statistically significant. Collectively, these in vivo results demonstrated that the anti‐CD22 mAb activated microglia and promoted microglial M1 polarization in EAE mice.

## 4 DISCUSSION

In the present study, we found increased levels of microglial CD22 following inflammation and EAE, and an in vitro study revealed that CD22 blockade promoted microglial M1 polarization. Furthermore, in vivo experiments demonstrated that CD22 blockade exacerbated EAE in mice and induced the polarization of microglia toward the M1 phenotype. Collectively, our findings demonstrated the critical role of CD22 in affecting the function of microglia following inflammation and EAE and indicated that microglial CD22 might serve as a target to limit neuroinflammation in EAE mice.

Consistently, several studies have demonstrated the upregulation of the Siglec family following inflammation.^22^, ^23^ Hauke Thiesler et al. reported that Siglec‐E in BV2 cells was significantly upregulated following inflammation and that LPS upregulated the expression of microglial Siglec‐E in a dose‐dependent manner in vivo. To date, no study has examined the expression level of microglial CD22 following inflammation and EAE. In our study, microglial CD22 was upregulated in a dose‐dependent manner after stimulation with LPS in vitro. In addition, an in vivo study revealed that CD22 was more strongly upregulated in the spinal cord than in the brain of EAE mice. Since myelitis is a characteristic pathological change in EAE mice, increased expression of microglial CD22 in the spinal cord may be associated with increased severity of inflammatory stimuli.

Previous studies have reported that the basal expression of microglial CD22 is relatively low.^18^, ^24^ It is enriched in mouse microglia 7 days after birth but is virtually absent in adult mice.^18^, ^24^ During the development of the CNS in neonatal mice, microglia can engulf apoptotic oligodendrocytes, and some scholars have speculated that CD22 in microglia may be used as a protective mechanism to inhibit the excessive phagocytosis of microglia through negative feedback regulation.^18^, ^24^ Interestingly, our research demonstrated that microglial CD22 could be dramatically induced following inflammatory stimuli and that elevated CD22 seems to inhibit the inflammatory response. Although no study has shown the functional role of microglial CD22 following inflammation and EAE, numerous studies have elucidated the anti‐inflammatory effect of Siglec on microglia. For example, Lexiao Li et al. reported that the knockdown of Siglec‐E could significantly promote the release of inflammatory cytokines in BV2 cells induced by LPS, suggesting that Siglec‐E in BV2 cells can inhibit the inflammatory response.^22^, ^23^ In addition, ectopic expression of Siglec‐11 in cultured mouse microglia inhibited the phagocytosis of neurons by microglia and reduced LPS‐induced inflammation in microglia, suggesting that microglial Siglec‐11 can protect neurons by inhibiting their own inflammatory response.^25^ Overall, their study showed that siglecs on microglia play a vital anti‐inflammatory and neuroprotective role in CNS diseases. Consistently, we found that CD22 blockade with cy34.1 mAb significantly exacerbated the clinical symptoms of EAE mice. Although CD22 was expressed in B cells, several studies have reported that cy34.1 mAb does not impair B cell function.^26^, ^27^, ^28^, ^29^

Spleen tyrosine kinase (SYK) instructs effector functions of CD22, and microglia rely on SYK to exert neuroprotection in models of MS.^30^, ^31^, ^32^, ^33^, ^34^ In addition, it was reported that phosphatidylinositol 3‐kinase (PI3K)/protein kinase B (AKT) signaling and mitogen‐activated protein kinase (MAPK) signaling also play important roles in modulating microglial activation and controlling the inflammatory responses of microglia.^35^, ^36^, ^37^, ^38^ The relationship of CD22 and PI3K/AKT and MAPK signaling should be further explored.

In this study, we provide evidence that CD22 is a key molecule governing the inflammatory function of microglia in EAE mice. However, there are several limitations in the present study. First, although in vitro and in vivo experiments have demonstrated that CD22 blockade can promote microglial M1 polarization, we did not construct microglia‐specific knockout mice to further verify the role of microglial CD22 in EAE. Second, determining the expression of CD22 on microglia and its functional role in patients is the first step toward clinical translation, and further studies are needed to determine whether MS patients have an elevated level of CD22. Finally, although H&E staining and immunofluorescence staining have revealed that CD22 blockade significantly increased inflammatory infiltration in the CNS of EAE mice, flow analysis should be performed to identify the subtype of infiltrating leukocytes in future studies.

## 5 CONCLUSION

Taken together, our study indicated that microglial CD22 was rapidly induced in EAE mice and that CD22 blockade aggravated the progression of EAE mice by promoting microglial M1 polarization, suggesting that CD22 may play an important role in the maintenance of immune homeostasis in EAE mice.

## AUTHOR CONTRIBUTIONS

All authors contributed to the study conception and design. WWX: Conceptualization, Methodology, Investigation, Writing – original draft. WK: Conceptualization, Methodology, Investigation. LH: Conceptualization, Methodology, Investigation. ZW: Visualization, Investigation. ZYZ: Writing – review & editing, Visualization, Investigation. SWB: Visualization, Investigation. JP: Visualization, Investigation. CX: Conceptualization, Supervision, Writing – review & editing. YTG: Conceptualization, Supervision. All authors read and approved the final manuscript.

## CONFLICT OF INTEREST STATEMENT

The authors have no relevant financial or non‐financial interests to disclose.

## Supporting information


Data S1.

## Data Availability

The data presented in this study are available on request from the corresponding author.
