# Supplementary material for: CD22 blockade aggravates EAE and its role in microglia polarization
Source: CNS Neurosci Ther. 2024 May 13;30(5):e14736. doi: 10.1111/cns.14736 (PMC11090149; doi:10.1111/cns.14736)
Supplement: Supplementary file 1 — Data S1. [file CNS-30-e14736-s001.pdf]

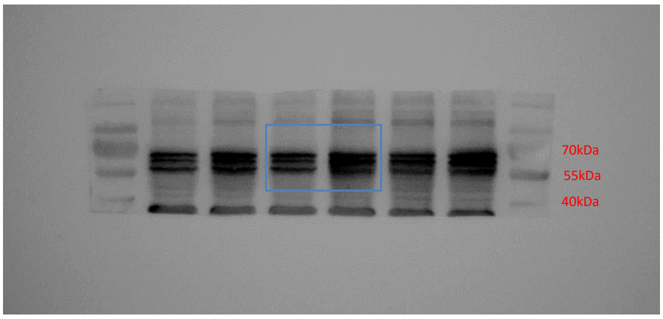

Full unedited gel/blot for Figure 4k

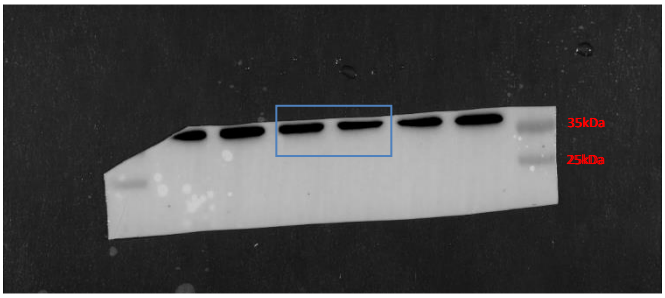

Full unedited gel/blot for Figure 4k

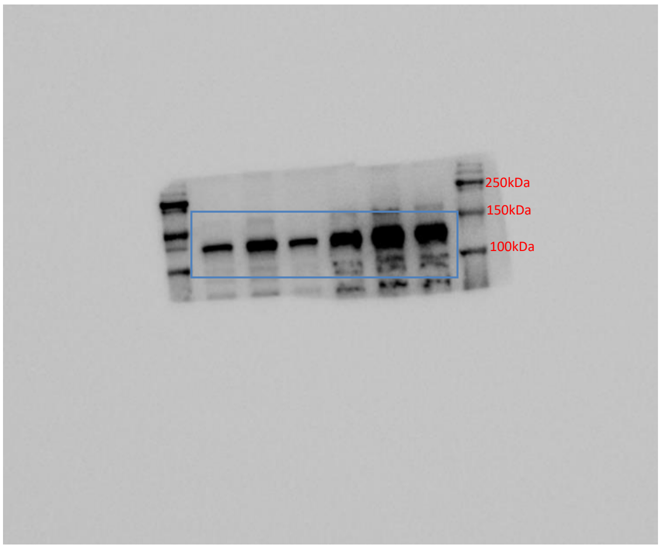

Full unedited gel/blot for Figure 6j

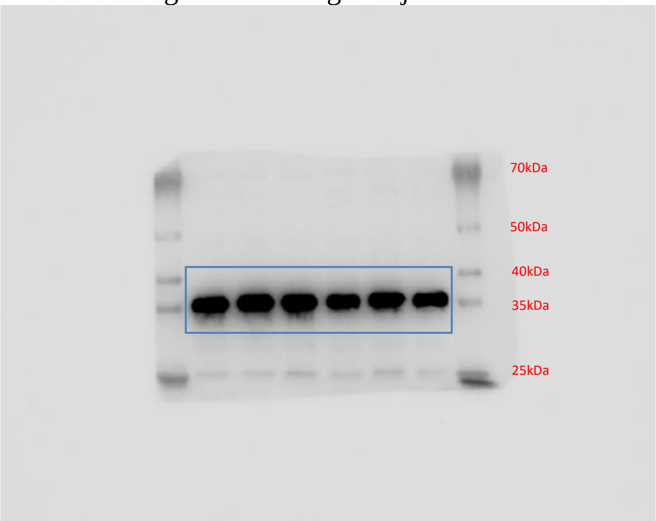

Full unedited gel/blot for Figure 6j
